# Clitoroplasty in the correction of clitoral hypertrophy: description of a technique that spares neurovascular structures

**DOI:** 10.1590/0100-6991e-20243781-en

**Published:** 2024-11-07

**Authors:** FELIPE LIMA JACOB, NYARA RODRIGUES CONDE DE ALMEIDA, LÍVIA GUERREIRO DE BARROS BENTES, RAFAEL SILVA LEMOS, JOÃO FILIPE DE SOUSA BARBOSA, KATIA SIMONE KIETZER, LUIS OTAVIO AMARAL DUARTE PINTO, EDSON YUZUR YASOJIMA

**Affiliations:** 1 - Universidade do Estado do Pará, Centro de Ciências Biológicas e da Saúde - Belém - PA - Brasil.; 2 - Universidade Federal do Pará, Instituto de Ciências Médicas - Belém - PA - Brasil.

**Keywords:** Clitoris, Hypertrophy, Surgery, Plastic, Anabolic Androgenic Steroids, Women’s Health, Clitóris, Hipertrofia, Cirurgia Plástica, Esteróides Androgênicos Anabolizantes, Saúde da Mulher

## Abstract

Clitoral hypertrophy is a condition that has a negative impact on a womans intimate life and can cause embarrassment and impact on her sexual life. The article describes a surgical technique of clitoroplasty performed with a 360° circumferential subcoronal incision only in the skin and Dartos tunica to avoid neuronal damage, followed by degloving to the base of the clitoris, preserving the dorsal neurovascular bundle. The body of the clitoris was amputated, preserving 0.5 cm of the stump of the clitoral shaft, and the glans was sutured to the rest of the remaining spongy tissue. Finally, it is noteworthy that there were no complaints of loss of sensitivity or sexual dysfunctions post-operatively.

## RESUMO

A hipertrofia do clitóris é uma condição que repercute negativamente na vida íntima da mulher e pode causar constrangimento e impacto na vida sexual. O artigo descreve uma técnica cirúrgica de clitoroplastia realizada com incisão subcoronal circunferencial de 360° apenas na pele e na túnica de Dartos para evitar danos neuronais, seguida do desenluvamento até a base do clitóris, preservando o feixe neurovascular dorsal. Prosseguiu-se com a amputação do corpo do clitóris, preservando 0,5 cm do coto da haste clitoriana, e a glande suturada ao restante do tecido esponjoso remanescente. Destaca-se, por fim, que não houve queixas de perda de sensibilidade ou disfunções sexuais no pós-operatório.

## INTRODUCTION

Clitoral hypertrophy is a condition with several negative repercussions on women’s self-esteem and sex life^1^ ^,^ ^2^. The excessive growth of this organ is called clitoromegaly, when exceeding 35mm^2-^ ^4^. Although it does not have major functional repercussions, the greatest impact is on the woman’s intimate life, which can generate embarrassment, psychological disorders, and restriction of sexual activities^4^ ^,^ ^5^. 

Clitoromegaly can be idiopathic or caused by different etiologies, intrinsic or acquired. Among the intrinsic causes are congenital adrenal hyperplasia, caused by enzymatic deficiencies that lead to excess production of adrenal steroids and stimulation of clitoral growth, exposure to maternal hormones, hormonal disorders at birth, polycystic ovary syndrome, and genetic alterations (mosaicism of sex chromosomes)^1^ ^-^ ^4^.

The main acquired cause of clitoromegaly is the use of exogenous anabolic androgenic steroids, such as nandrolone, anadrol, methanolone, among others. This effect is caused by the action of synthetic testosterone derivatives, the main components of anabolic steroids, stimulating hair, changes in voice pattern, muscle hypertrophy, and virilization^5^. Hypertrophy of the clitoris can be irreversible and cause discomfort, pain, and changes in sensitivity in the region. The percentage of women who experience this side effect varies according to the dose, time, and type of anabolic steroid used, and can occur in more than half of users^6^. 

In recent years, there has been an increase in the number of cases of clitoromegaly, related to the increase in the number of anabolic steroid users^6^ ^,^ ^7^. Among the main reasons for this increase are the change in the standard of beauty and the growth of gym culture (overvaluation of athletic bodies), muscle mass gain and fat loss, stagnation of muscle development, in addition to improved physical performance^4^ ^,^ ^6^. Other factors may also be related, such as eating disorders, anorexia, obsessive compulsive symptoms, psychological trauma, and body dysmorphia^4^. 

In this context, despite the relevance of the theme for the self-esteem and quality of life of patients with clitoromegaly, there is still no consensus in the literature on the best surgical technique for clitoral reduction. Thus, the objective of this study is to describe a surgical technique that spares neurovascular structures for the correction of clitoromegaly due to use of anabolic steroids.

## TECHNIQUE AND RESULTS

**Figure 1:**
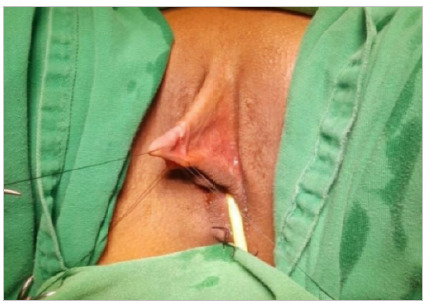
Repair stitch through the glans.

**Figure 2:**
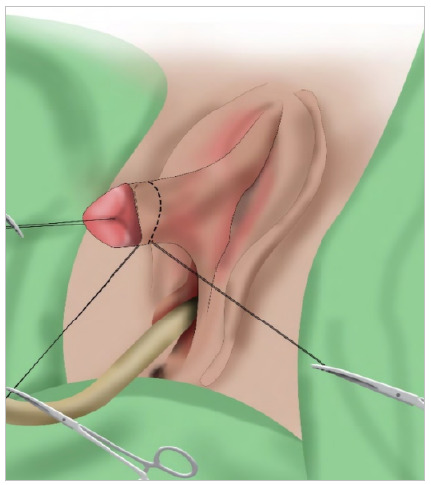
Repair stitch on the glans, schematic drawing.

After antisepsis, asepsis, and placement of sterile drapes, the patient was anesthetized with spinal anesthesia and positioned in simple lithotomy. Next, a nº 14 Foley catheter was introduced through the urethra and a repair stitch was made on the glans with 4-0 nylon surgical thread to facilitate the exposure of the clitoris (Figures 1 and 2).

**Figure 3:**
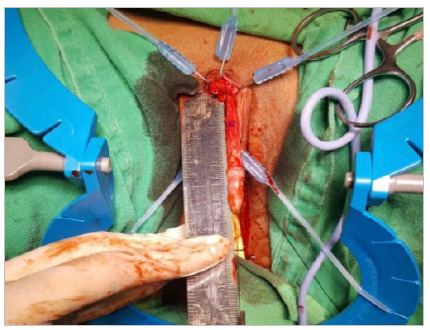
Degloved clitoris measuring 6.5 cm.

Next, a 360° circumferential subcoronal incision was made only in the skin and tunica dartos to avoid neuronal injury, and then degloving was conducted till the base of the clitoris. At that time, we found that the length of the clitoris under maximum traction was 6.5cm (Figure 3).

**Figure 4:**
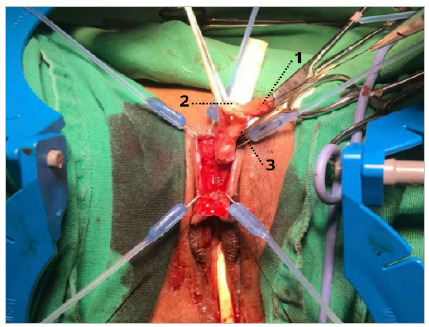
Release of the dorsal nerve vascular bundle. 1 - Glans of the clitoris. 2 - Dorsal vascular-nervous bundle of the clitoris. 3 - Corpus cavernosa of the clitoris..

**Figure 5:**
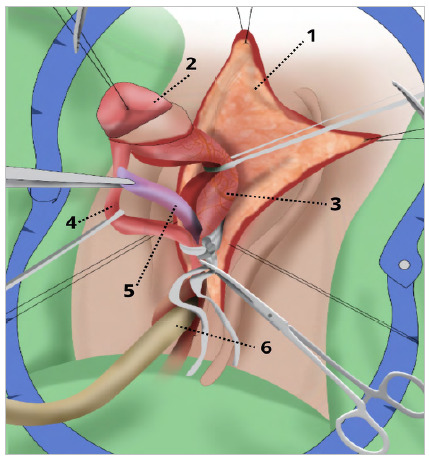
Release of the dorsal nerve vascular bundle, schematic drawing. 1 - Flap of skin from the degloved clitoris. 2 - Glans of the clitoris. 3 - Dorsal vascular nervous bundle clitoris. 4 - Urethral plate. 5 - Corpus cavernosa of the clitoris. 6 - Bladder probe.

After degloving, a tourniquet was performed using a Penrose nº 0 drain at the base of the clitoris for hemostatic control, in addition to facilitating the visualization of the structures (Figures 4 and 5). Then, from incisions in Buck’s fascia at 5 a.m. and 7 a.m., the dorsal neurovascular bundle (DNVB) was released, in which the tunica albuginea of the clitoris was kept in the position from 10 a.m. to 2 a.m. to decrease the risk of injury to DNVB. 

**Figure 6:**
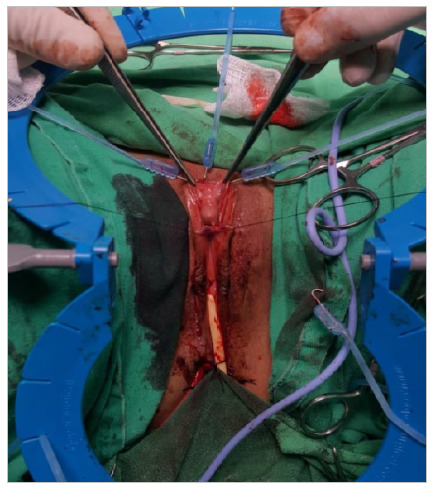
Fixation of the glans in the corpora cavernosa.

**Figure 7:**
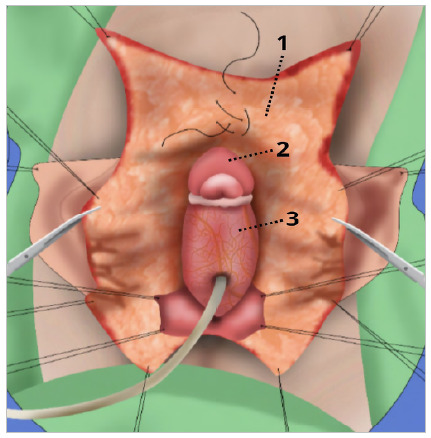
Fixation of the glans in the corpora cavernosa, systematic design. 1 - Flap of skin from the degloved clitoris. 2 - Glans of the clitoris. 3 - Urethral plate.

After the release of the DNVB, the glans/DNVB complex was detached from the rest of the clitoris and the excess corpora cavernosa was amputated, leaving a stump of approximately 0.5cm, which was sutured with Vycril 3-0 surgical thread. Next, the glans was fixed to the remaining corpus cavernosum and the DNVB was fixed under the subcutaneous tissue near the pubic symphysis (Figures 6 and 7).

**Figure 8:**
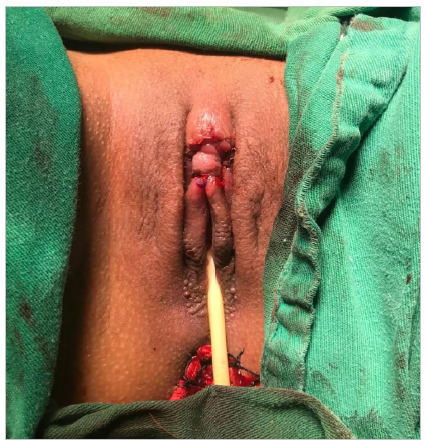
Reconstruction of the clitoral hood and labia minora.

**Figure 9:**
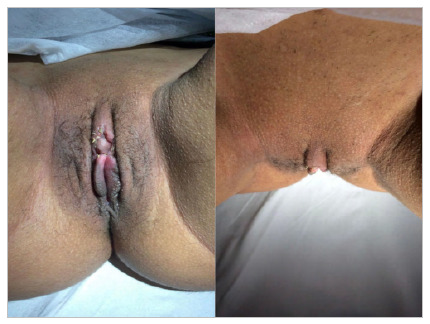
Appearance after one month postoperatively.

Finally, the previously degloved excess skin was sectioned and the synthesis by planes was performed with Vycril Rapid 4-0 surgical thread at separate stitches for reconfiguration of the labia minora and clitoral hood (Figures 8 and 9).

The patient was discharged from the hospital 48 hours later, with subjective reports of paresthesia and discomfort in the region. The urinary catheter remained installed for 14 days in the postoperative period, associated with a perineal compressive dressing, with routine changes, without the need to remove surgical stitches, due to the use of absorbable suture. The return to sexual activities was allowed two months after surgery.

After follow-up for two months postoperatively, there were no reports of loss of sensation or sexual dysfunctions.

## DISCUSSION

The cult of the body and the premise of adapting to established beauty standards have led to an increasing number of women making use of anabolic-androgenic hormones for aesthetic purposes with quick results^4^ ^,^ ^8^. Indiscriminate use by women, especially related to increased body mass and sports performance, can lead to important undesirable masculinizing effects, such as increased body hair, voice changes, reduced breast volume, clitoral enlargement, gonadal dysfunction, abnormal uterine bleeding, and infertility, changes that affect women’s self-esteem and social life^4^ ^,^ ^8^ ^-^ ^10^. In this scenario, considering that there is not enough clinical and scientific confirmation regarding the benefit and safety that outweigh the harms of the practice, in 2023, the Federal Council of Medicine of Brazil prohibited the use of formulations of anabolic steroids or androgenic hormones for aesthetic purposes or to improve sports performance^11^. 

Due to the increasingly widespread use of anabolic steroids and the increase in the frequency of their undesirable masculinizing effects, clitoral correction surgery has required increments and updates in recent decades, to meet the aesthetic and functional postoperative results, preserving the sexual sensitivity of the organ^5^ ^,^ ^7^ ^,^ ^13^. For this, it is crucial the surgical shortening of the corpora cavernosa, preserving the neurovascular supply to the glans^5^ ^,^ ^7^ ^,^ ^14^. 

First practiced by the clitorectomy technique, performed by Young^15^ in 1934, this rudimentary approach added unsatisfactory postoperative results, such as irreversible loss of clitoral sensitivity, decreased orgasm, and painful erections, factors that led to the abandonment of surgical techniques of total and partial clitorectomy^2^ ^,^ ^15^. In this sense, hypertrophied clitoris reconstruction surgery has gained technical improvements supported by a more in-depth knowledge of urogenital anatomy, which proposed minimal effects on its functionality, ensuring quality of life for the operated women^2^ ^,^ ^5^ ^,^ ^7^ ^,^ ^13^ ^,^ ^14^. Currently, surgical approaches of reduction clitoroplasty are aimed at preserving the dorsal neurovascular pedicle, originating from the pudendal nerve, and the extensive nerve network around the corpora cavernosa, aiming to maintain genital aesthetics and preserve sexual functions in the long term^2,^ ^5^ ^,^ ^7^ ^,^ ^14^.

In this context, since the manipulation of such structures requires improvement of technical skills and manual dexterity, anatomical knowledge of the neurovascular structures of the clitoris is of paramount importance for surgical success^2^ ^,^ ^5^ ^,^ ^7^ ^,^ ^14^ ^,^ ^17^. The technique addressed in the study consisted of dissection of the neurovascular bundle associated with preservation of the tunica albuginea in the dorsal region of the clitoris after the release of Buck’s fascia and preservation of the clitoral glans. This approach aims to reduce the chances of iatrogenic injury to the bundle, which could result in postoperative paresthesias, ischemia, and necrosis of the clitoris, in the same way as it has been used by other authors^3^ ^,^ ^5^ ^,^ ^16^ ^,^ ^17^.

The aesthetic and functional results of the approach with separation and preservation of the neurovascular pedicle are superior, since they added minimal long-term adverse effects, ensuring minimal loss of clitoral sensitivity, sexual intercourse, and non-painful erections, in addition to favorable aesthetic results^2^ ^,^ ^3^ ^,^ ^5^ ^,^ ^7^ ^,^ ^14^ ^-^ ^17^. Comparatively, in the study by Sönmezer (2023), all women who underwent clitoroplasty surgery with preservation of the neurovascular bundle presented, at the end of 24 months of observation, preservation of clitoral sensitivity. Similarly, in the present study, after two months postoperatively, there were no reports of loss of clitoral sensation, pain, or local edema, even after sexual intercourse. 

In view of this, it is understood that clitoroplasty using the neurovascular conservation technique restores normal anatomy, allows a favorable aesthetic result and, above all, reduces the chances of impaired sexual function, reducing impacts on the social life of operated patients^3^ ^,^ ^17^ ^,^ ^18^. The choice of operative technique should always respect the individuality of each case and the professional’s experience. 

Complications may include loss of clitoral sensation, ischemia, and necrosis of the clitoral glans. Although possible, these consequences are less frequent in the surgical technique described, since care is taken to preserve the dorsal neurovascular bundle of the clitoris, which reduces the chances of such complications^5^ ^-^ ^18^.

Although clitoromegaly is an increasingly common complaint in the offices of gynecologists, urologists, and plastic surgeons, there is no standardization of the surgical approach, given the limitation of case studies in the literature^2^ ^,^ ^5^ ^,^ ^14^ ^,^ ^16^ ^,^ ^17^. Hence the importance of more publications on this topic, necessary for a thorough and descriptive understanding of urogenital anatomy and long-term anatomical-functional evaluation of patients in the postoperative period, as well as for enabling greater discussion among specialists about the different surgical techniques and their impacts on patients’ health.

## CONCLUSION

Clitoromegaly has become an increasingly common condition, given the growth of indiscriminate use of anabolic-androgenic hormones. Currently, there is no standardization in the literature regarding the surgical reconstruction technique in clitoroplasty. However, the use of neurovascular bundle release techniques seems promising, due to the lower risk of iatrogenic injury, in addition to enabling significant aesthetic and functional results, with minimal implications for clitoral sensitivity.
